# Physical Activity of German Children and Adolescents 2003–2012: The MoMo-Study

**DOI:** 10.3390/ijerph14111375

**Published:** 2017-11-11

**Authors:** Steffen C. E. Schmidt, Annette Henn, Claudia Albrecht, Alexander Woll

**Affiliations:** Institute of Sports and Sports Science, Karlsruhe Institute of Technology, 76131 Karlsruhe, Germany; Annette.Henn@kit.edu (A.H.); Claudia.Albrecht@kit.edu (C.A.); Alexander.Woll@kit.edu (A.W.)

**Keywords:** physical activity, sports, playing outside, children, adolescents, MoMo

## Abstract

Organized and unorganized physical activity (PA) hold an important role in the daily life of children and adolescents. Regular representative tracking of PA in different settings is important to evaluate social trends and implemented interventions. In this paper, representative PA data of German children and adolescents from the MoMo Baseline-Study (2004, *n* = 4528) are compared to those of Wave 1 (2010, *n* = 3994). Participants aged 4–17 were drawn out of 167 sample points in Germany and the data was weighted to ensure representativeness. PA was measured via questionnaire and was differentiated between organized (sports clubs and schools) and unorganized (unorganized sports and playing outside). Organized PA in extracurricular activities and sports clubs increased by eight percent, while unorganized PA decreased by seven percent. In addition to sports clubs, schools became a more prevalent setting for participation in physical activity in Germany.

## 1. Introduction

The health benefits of physical activity (PA) during childhood and adolescence are well documented [1], and an active lifestyle during childhood is an important factor in preventing disease in adulthood [2,3,4]. The World Health Organization (WHO) therefore promotes PA as part of a healthy lifestyle [5]. However, in a global study about the PA of children and adolescents, Tremblay and colleagues reported low levels of PA in 10 out of 15 participating countries and noted that there is strong evidence for a “childhood physical activity crisis” [6]. In another study among 105 different countries, Hallal and colleagues found that only 20% of 13- and 15-year-olds reported at least 60 min of daily moderate- to vigorous-intensity PA [7]. Although numbers of physically inactive children and adolescents vary widely across nations, there is consensus about some variables that are consistently associated with children’s PA. Some of those variables, such as perceived barriers, perceived activity competence, program/facility access, parental support, and time spent outdoors [8] clearly identify a child’s environment as a crucial factor for their PA. Among the 15 investigated countries in Tremblay’s study, only New Zealand reported high values of unorganized PA, with an average of 78 min per day [9]. A decline in unorganized outdoor PA is reported for example in Canada [10], Finland [11], and Germany [12]. In addition, many nations report a decline in active transport [7].

These circumstances offer a variety of possibilities for intervention, but in order to properly intervene and convince politicians and decision-makers, it is important to track and report detailed information about PA, especially in different settings and from representative samples.

In Germany, the PA of children and adolescents is tracked by the Motorik-Modul Study (MoMo) [13]. In this paper, we report the MoMo PA data of two representative cross-sectional samples of children and adolescents, 2003–2006 (Baseline) and 2009–2012 (Wave 1), and discuss changes in different settings.

## 2. Materials and Methods

### 2.1. Sample and Repressentativeness

The MoMo Study is a nationwide study on physical fitness and physical activity in German children and adolescents, and part of the German Health Interview and Examination Survey for Children and Adolescents, KiGGS [13,14]. To ensure a diverse sample of German children and adolescents, a nationwide, stratified, multi-stage sample with three evaluation levels was drawn [15]. First, a systematic sample of 167 primary sampling units was selected from an inventory of German communities that were stratified according to the BIK classification system that measures the level of urbanization and the geographic distribution [14]. The probability of any community being picked was proportional to the number of inhabitants younger than 18 years in that community. Second, an age stratified sample of 28,400 randomly selected children and adolescents was drawn from the official registers of local residents. Ultimately, 17,641 participated in the KiGGS Baseline study (62.1%). At the second measurement point (KiGGS Wave 1 study), 12,368 children and adolescents participated (4455 newly invited and 7913 from Baseline, response rates of 38.8% and 72.9%, respectively) [16]. Those two samples built the population for the MoMo subsample. 7866 participants from KiGGS Baseline were randomly assigned to the MoMo Baseline, and 6076 from KiGGS Wave 1 were randomly assigned to MoMo Wave 1. The final number of participants aged 4–17 years in MoMo was *n* = 4528 at Baseline (57.6%) and 3994 in Wave 1 (65.7%). In order to obtain final response rates, the initial KiGGS response and the later MoMo response were multiplied, resulting in final response rates of 35.8% for MoMo Baseline and 36.4% for MoMo Wave 1.

A weighting procedure was used to account for potential bias in outcome variables caused by selective unit nonresponse [15]. In the first step, inverse probability weights were applied via logistic regression to eliminate differences in outcome variables between the MoMo subsample and the weighted representative KiGGS sample. In the second step, the MoMo subsample was stratified using data of the German Micro Census 2004 and 2010 to ensure representativeness of the target population (German children and adolescents aged 4–17 years) regarding sex, age, region, migration background, and education level.

MoMo Baseline data was collected between 2003 and 2006, and MoMo Wave 1 data between 2008 and 2012. Parents and adolescents were invited to examination rooms at central locations within close proximity to their homes in the 167 cities and municipalities, in which the study was conducted. They gave written informed consent and then answered questionnaires in the presence of a qualified interviewer on site.

### 2.2. PA Assessment

The MoMo Physical Activity Questionnaire (MoMo-PAQ) was used to assess self-reported habitual PA in adolescents in different settings (sports clubs, leisure time, school, daily activities and overall physical activity) [17]. The MoMo-PAQ consists of 28 items and measures frequency, duration, intensity, and setting of PA in a normal week, without a defined reference period. Data obtained with the MoMo-PAQ are sufficiently reliable (test-retest reliability: *ICC* = 0.68), but correlation coefficients with accelerometry data are rather low (*r* = 0.29) [17].

PA in schools was assessed by two items about the frequency (times per week) of 45 min classes in curricular and extracurricular sports activities. Calculated total minutes were multiplied with a correction factor of 8.5/12 to compensate for vacations.

Sports club physical activity was assessed by four items: type of sports club activity, duration (minutes per session), frequency (times per week) of each activity, and time throughout the year (months per year) of each activity. From those items, minutes per week at sports clubs were calculated.

Unorganized, leisure-time sports activity was assessed by three items: type, duration (minutes per week), and time throughout the year (months per year). These items were combined in an index reflecting minutes per week at leisure-time unorganized sports activities.

Unorganized playing outside was assessed by an 8-scaled item about days per week in which the child plays outside (“How often do you normally play outside during a week (for example: playing tag, skipping rope, or going to the swimming pool)”), and an item about minutes spent on average during one of those days.

### 2.3. Statistics

All of the statistical tests were conducted using IBM SPSS 24 (IBM Corporation, Armonk, NY, USA). Statistically significant differences between Baseline and Wave 1 were determined via 95% confidence intervals (95% CI) for complex samples. The SPSS complex sample procedure accounts for weighting and the fact that MoMo is a cluster sample of 167 sample points in Germany. *p* < 0.05 was assumed when both 95% CIs did not include the mean of their counterpart. Unless otherwise specified, data is presented weighted to ensure representativeness [15]. Two sample Student’s *t*-Tests and CHI^2^-Tests were used to determine significant gender differences.

## 3. Results

The MoMo-PAQ differentiates PA into different settings. In the following PA in schools section, organized PA in sports clubs as well as PA in unorganized sports and PA in the form of playing outside is reported.

### 3.1. PA in Schools

During school time, children and adolescents are required to participate in curricular sports. However, compulsory amounts differ between states and schools. In addition, in most schools children and adolescents get the opportunity to participate in extracurricular sports activities. Table 1 shows the weekly hours spent in curricular sports at Baseline and Wave 1, as well as the percentage of children and adolescents engaging in extracurricular sports, and if they do, the hours per week.

Time spent in curricular sports slightly decreased in preschool children and increased for children aged 6–13. Overall, a slight but significant increase of 2.9 min/wk was observed in curricular sports. Curricular sports activity increased with age until the age of 13, and then decreased in the higher classes. In addition, boys showed slightly more time spent in curricular sports than girls (Baseline: *p* = 0.02, T = 2.39; Wave 1: *p* = 0.04, T = 1.97). 

Besides curricular sports, participation and time spent in extracurricular sports nearly doubled during the observed time span. This increase in extracurricular sports attendance is attributed to the 6- to 10-year-olds with 9.8% (95% CI: 8.0–12.0) at Baseline and 24.6% (95% CI: 20.8–28.9) at Wave 1 and to the 11- to 13-year-olds with 14.1% (95% CI: 11.0–17.8) and 21.5% (95% CI: 17.5–26.1), respectively. 14- to 17-year-olds did not significantly increase their participation in extracurricular sports from Baseline to Wave 1. Finally, a small difference favoring boys in extracurricular sports activities was observed, which is statistically significant for Wave 1 (Baseline: *p* = 0.36, T = 0.91; Wave 1: *p* = 0.03, T = 2.20).

### 3.2. PA in Sports Clubs

In Germany, sports clubs are traditionally well attended. Therefore, they play an important role in children’s and adolescents’ PA and PA education. Table 2 shows the percentage of children and adolescents who are members in sports clubs and the minutes per week that are spent in total among all study participants.

The number of children and adolescents engaging in PA in sports clubs increased significantly from 53.5% (95% CI: 52.9–57.9) to 61.5 (95% CI: 58.5–63.7). Concurrent time spent with PA in sports clubs significantly increased 22.3 min/wk from 89.4 min/wk (95% CI: 82.6–96.2) to 111.7 min/wk (95% CI: 105.2–118.2). Participation and time spent in sports clubs increased among all of the age groups and both sexes. Overall participation peaks between the ages of 6 and 13, and time spent with PA in sports clubs peaks at the age of 14–17. Boys show overall higher numbers for participation (Baseline: *p* < 0.01, Chi^2^ = 50.37; Wave 1: *p* < 0.01, Chi^2^ = 37.54), as well as more time spent with PA in sports clubs (Baseline: *p* < 0.01, T = 11.05; Wave 1: *p* < 0.01, T = 10.47). However, interestingly, girls show a numerically higher amount of sports club PA at the age of 4–5 at Baseline and statistically significantly more in Wave 1 (Baseline: *p* = 0.26, T = 1.14; Wave 1: *p* = 0.01, T = 2.54).

### 3.3. Unorganized PA

Besides organized PA in school and sports clubs, unorganized PA including unplanned PA with friends, playing outside and working out at home, outside, or in gyms defines children’s and adolescents’ daily PA. Table 3 shows time spent in unorganized sport activities and days per week with playing outside for the different age groups and sexes.

Overall time spent in unorganized sports activity significantly decreased by 21.1 min/week, from 83.1 min/week (95% CI: 77.2–90.0) to 62.1 min/week (54.9–69.3). Both sexes and every age group are affected. Aside from sports activity, unorganized playing outside only slightly decreased overall, with no change in preschool children, a slight increase in primary school, and a decrease among 11- to 17-year-olds. At Baseline, boys showed higher amounts of unorganized sports activity in every age group (*p* < 0.01; T > 2.43) and engaged in playing outside more often (*p* < 0.01, T > 3.1), except for 11- to 13-year-olds (*p* = 0.25, T = 1.14). At Wave 1, no gender differences were found for 4- to 5- or 6- to 10-year-olds in unorganized PA and playing outside (*p* > 0.49, T < 0.69). However, at the age of 11–13, boys spent more time in unorganized PA (*p* < 0.01, T = 2.94), but not in playing outside (*p* = 0.13, T = 1.53). At the age of 14–17, boys spent more time in unorganized PA (*p* < 0.10, T = 2.60) and played outside more often (*p* < 0.01, T = 4.65).

## 4. Discussion

### 4.1. Overall Findings

The study shows an increase in organized physical activity paralleled by a decrease in unorganized physical activity among German children and adolescents ages 4–17 from 2004 to 2010. The overall increase in sports club PA of 22.3 min/week is almost entirely covered by a decrease of unorganized sport activity of 21.1 min/wk. However, due to positive trends in school-based PA of 2.9 min/week in curricular sports and 6.1 min/week in extracurricular sport activities, an overall positive trend for German children and adolescents’ PA can be seen.

### 4.2. PA in School

Time spent in curricular sports increased significantly by 2.9 min/week, but we do not derive meaningful practical relevance from this slight increase. Curricular sports turned out to stay mainly unchanged over the observed time span. However, time spent in extracurricular sports nearly doubled during the observed time span due to a large increase among 6- to 13-year-olds. There is broad agreement that the main reason for this trend in Germany is the implementation of daytime schools in the educational offering. In daytime schools, extracurricular courses, including sports, are offered more frequently. In recent discussions, authors mentioned that these offerings could reduce PA in sports clubs [18]. There is no evidence for this hypothesis in our data. On the contrary, correlation coefficients (unweighted) between time spent in extracurricular sport activities and sports clubs tend to be positive (6–10 years: *r* = 0.09, *p* < 0.05; 11–13 years: *r* = 0.05, *p* = 0.31; and, 14–17 years: *r* = 0.11, *p* < 0.01).

Boys reported slightly higher amounts of curricular and extracurricular PA in school, but mean differences of about 2.2 min/week among genders should be viewed as having low to no practical relevance. On the other hand, the rather small gender differences in school-based PA when compared to larger differences in sports club PA among adolescents ages 11–17 offer promising approaches to promote PA among girls in the school setting.

### 4.3. PA in Sports Clubs

61.5% of children and adolescents reported PA in sports clubs at Wave 1. An increase of 8% as compared to 53.5% at Baseline is confirmed by official numbers from the German Olympic Sports Confederation [19] and independent studies about German sports clubs [20]. The German Olympic Sports Confederation reported an increase in sports club membership of 5.6% in 15- to 18-year-olds and 13.0% in 7- to 14-year-olds from 2000 to 2010 [19]. However, the absolute number of sports club members in children and adolescents declined [19] due to declining birth rates. Interestingly, girls were more likely to engage in sports club PA than boys at a preschool age. Only in primary school do boys heavily increase their sports club participation and outnumber their female counterparts. This phenomenon is confirmed by German [21], as well as international, studies [22,23]. Finally, a decline in participation in middle and late childhood was observed at both measurement points, but due to higher frequency and duration of training, the overall time spent in sports club PA did not decrease with increasing age. A decline in organized PA participation with age during childhood is also reported in Canadian studies [24,25,26].

### 4.4. Unorganized PA

The MoMo study shows that in Germany, unorganized sports activity, as well as playing outside, decreased between 2003 and 2012. Canadian studies also reported a downwards trend in active play [10], but authors declared that the causal for this is yet unknown. Tremblay et al. [6] state that it is commonly believed in developed countries that active, unstructured play is decreasing for a variety of reasons, including increased screen time, safety concerns (e.g., traffic, stranger danger), emphasis on organized youth sports, and parental work. Nevertheless, in a recent study across 25 countries, “playing with friends” was the top response (30%) [27], and data from the United States between 1981 and 2003 showed that “playing” was the most common pastime after television viewing among 6- to 12-year-olds [28].

To date, many voices blame screen time as one of the main causals for less unorganized sport activity and playing outside. However, contrary to productive sedentary time like doing homework, reading books or working on the computer, TV viewing and gaming showed no relation to PA in a Canadian study [29]. Although a relationship between PA and screen time seems plausible, recent studies suggest that correlation between PA and screen time is low to non-existent [30,31].

Conforming with the aforementioned studies, no to low correlation (unweighted) between screen time and PA could be found in the MoMo data (*r* < −0.25). The highest correlation between screen time and PA was found for computer usage and PA in sports clubs among 6- to 10-year-olds (*r* = −0.12, *p* < 0.01) and 11- to 13-year-olds (*r* = −0.16, *p* < 0.01), and computer usage and playing outside in 6- to 10-year-olds (*r* = −0.12, *p* < 0.01), 11- to 13-year-olds (*r* = −0.25, *p* < 0.01) and 14- to 17-year-olds (*r* = −0.11, *p* < 0.01). TV and console usage showed a correlation of <0.11 or no significant correlation with PA in sports clubs, unorganized PA, and playing outside. These results are in line with a previous study with MoMo Wave 1 data using cluster-analyses to identify active/sedentary behavioral patterns [32]. The study showed patterns with high media use and low PA in adolescents, but at the same time adolescents with high PA also had considerable amounts of media use, and the lowest media use was accompanied with very low PA. An explanation for this could be an underlying urge to move among children and adolescents that is not suppressed by media use, but is satisfied by any form of PA. The thesis of a low relationship between PA and screen time is also supported by a German study that showed an increase in screen time due to computer usage, but at the same time also a slight increase in PA from 2002 to 2010 [33]. A declining trend in TV usage, but increasing computer screen time was verified in an international study across 30 nations and speaks for an internal cannibalization among competing sedentary behaviors [34]. Most of the aforementioned studies are observational, but to uncover causal effects, experimental designs are needed. A recent study with a screen-time-decreasing intervention reported no increase in PA after a meaningful reduction of screen time in children [35].

Although studies show that physical activity, especially unorganized activity, is an effective way to decrease obesity in children [36], active play is likely of light intensity, and to date, the importance of light and/or incidental PA, especially in the form of active play, is largely unknown [6]. Therefore the fact that unorganized PA decreases while organized PA increases is not an unmitigated negative development, though we strongly recommend further tracking and observation of this trend.

### 4.5. Strength and Limitations

The present study is limited to its observational nature and we do not intend to infer causality from paralleled trends or significant correlations. The main goal of MoMo was to track and report PA and fitness of children and adolescents in a nationwide sample, and significant effort was put into collecting representative data from 167 sample points all over the country.

PA was assessed by self-reports. This method has various limitations, including recall bias and social desirability. Measuring PA by objective methods such as accelerometers is more accurate in most types of PA, but is always limited to a short time interval, and unless a diary is added, the setting in which the PA took place is not captured. A relationship of only *r* = 0.29 between self-reported PA and accelerometer data was found in a validation study [17]. However, since accelerometers capture any form of PA in a specific time frame, the correlation with self-reported habitual PA in specific settings is expected to be restricted, even when summarized. An accelerometer is also a very responsive tool towards socially desired behavior, and children drop it for some sports like swimming, martial arts, and sometimes even curricular sports in school. Therefore, we do not necessarily conclude a low validity of self-reports from low correlation with accelerometer data, but we have to state that the data is not entirely comparable. Using a questionnaire offered the chance to assess different types of exercise as well as other PA parameters like setting and sports club membership during a normal week, even when the person is, for example, on vacation or ill. However, to further improve the quality of the MoMo physical activity data, accelerometer measurements have been included at Wave 2 (2015–2017).

## 5. Conclusions

The representative data from the MoMo study about children and adolescents in Germany shows an increase in organized PA (school & sports club), paralleled by a decrease in unorganized PA (unorganized sports & playing outside) between 2004 and 2010. In sum, a slight increase in PA has been found. MoMo is an observational study and we cannot make statements about the underlying cause for this positive trend, but the increasing amount of time spent in organized PA speaks in favor of PA interventions and offers.

Finally, the declining amount of time spent in unorganized PA should be further observed. On one hand, replacing unorganized PA with organized PA most likely increases the quality of movement time, but on the other hand, unstructured PA is an important part of children’s development and should not be replaced entirely. Though the fact that playing outside did not decrease among the 4- to 10-year-olds provides an overall positive picture for recent PA trends among German children and adolescents.

Looking back at the results that we found in the MoMo study, we strongly encourage further tracking of children and adolescents’ PA with self-reports in large epidemiological studies, but also recommend the usage of accelerometers in controlled experimental designs to evaluate PA interventions and uncover causal effects in the field of PA behavior. When an additional control group is examined, most of the disadvantages of accelerometers vanish. Accelerometers are therefore an important tool to further investigate the relationship between PA and sedentary behavior in experimental designs.

## Figures and Tables

**Table 1:** Physical activity (PA) of German children and adolescents in setting school.

		MoMo Baseline (2003–2006) *n* = 4528			MoMo Wave 1 (2009–2012) *n* = 3994		
		Curricular Sports	Extracurricular Sports		Curricular Sports	Extracurricular Sports	
Age (Years)	Sex	Min/Week ^1^ Mean ± SD	Min/Week ^1^ Mean ± SD	Percentage Engaging	Min/Week ^1^ Mean ± SD	Min/Week ^1^ Mean ± SD	Percentage Engaging
4–5	m	* 45.1 ± 35.1	-	-	* 37.7 ± 22.2	-	-
	f	* 49.3 ± 41.3	-	-	* 43.3 ± 26.8	-	-
	Ø	* 47.1 ± 38.3	-	-	* 40.5 ± 24.7	-	-
6–10	m	* 79.5 ± 27.4	* 5.1 ± 18.3	* 8.6%	* 84.8 ± 30.9	* 15.3 ± 27.8	* 22.3%
	f	* 76.2 ± 27.0	* 4.0 ± 17.4	* 11.0%	* 80.3 ± 26.9	* 11.9 ± 24.7	* 26.8%
	Ø	* 77.9 ± 27.2	* 4.6 ± 17.8	* 9.8%	* 82.6 ± 29.1	* 13.6 ± 26.3	* 24.6%
11–13	m	* 81.9 ± 26.6	* 8.2 ± 24.9	* 14.1%	* 88.4 ± 35.6	* 13.0 ± 30.8	* 24.0%
	f	* 78.9 ± 24.3	* 8.4 ± 24.7	* 14.0%	* 86.3 ± 31.1	* 14.2 ± 27.3	* 19.1%
	Ø	* 80.5 ± 25.6	* 8.3 ± 24.8	* 14.1%	* 87.3 ± 33.5	* 13.6 ± 29.1	* 21.5%
14–17	m	67.5 ± 28.9	6.7 ± 22.2	10.8%	69.0 ± 29.4	8.8 ± 25.0	9.3%
	f	65.0 ± 26.1	6.0 ± 21.7	11.2%	66.8 ± 27.9	5.7 ± 18.8	13.8%
	Ø	66.3 ± 27.6	6.3 ± 21.9	11.0%	67.9 ± 28.7	7.3 ± 22.2	11.6%
4–17 ^2^	m	72.2 ± 30.9	* 6.4 ± 21.5	* 10.8%	75.0 ± 34.6	* 12.4 ± 27.8	* 18.2%
	f	* 70.0 ± 29.7	* 5.8 ± 21.0	* 11.8%	* 72.9 ± 31.3	* 10.3 ± 23.8	* 20.1%
	Ø	* 71.1 ± 30.3	* 6.1 ± 21.3	* 11.3%	* 74.0 ± 33.0	* 11.4 ± 25.9	* 19.2%

^1^ Minutes are multiplied by a factor of 8.5/12 to compensate for holidays; ^2^ Age 6–17 for extracurricular sports; * Significant difference (*p* < 0.05) between Baseline and Wave 1. SD: standard deviation, m: male, f: female, Ø: all.

**Table 2:** PA of German children and adolescents in setting sports club.

		MoMo Baseline (2003–2006) *n* = 4528		MoMo Wave 1 (2009–2012) *n* = 3994	
Age (Years)	Sex	Min/Week Mean ± SD	Percentage Members	Min/Week Mean ± SD	Percentage Members
4–5	m	36.3 ± 54.2	43.3	41.5 ± 54.7	48.6
	f	* 41.3 ± 51.3	* 50.3	* 54.6 ± 63.1	* 62.7
	Ø	38.8 ± 52.8	46.8	49.2 ± 59.2	55.4
6–10	m	* 93.2 ± 96.4	65.6	* 118.0 ± 118.0	71.6
	f	* 57.8 ± 76.4	* 51.4	* 80.8 ± 100.4	* 60.7
	Ø	* 76.0 ± 89.0	* 58.7	* 99.3 ± 111.3	* 66.2
11–13	m	133.0 ± 144.0	64.3	147.1 ± 149.0	67.6
	f	* 79.7 ± 112.1	* 49.1	* 127.9 ± 142.8	* 65.6
	Ø	* 107.2 ± 132.2	* 56.9	* 137.8 ± 146.2	* 66.6
14–17	m	* 138.3 ± 177.5	* 54.0	* 182.5 ± 187.7	* 66.6
	f	83.4 ± 131.3	43.3	82.2 ± 131.0	42.2
	Ø	* 111.5 ± 159.0	48.8	* 134.2 ± 170.3	54.7
4–17	m	* 109.2 ± 138.9	* 58.7	* 133.9 ± 150.7	* 66.1
	f	* 68.6 ± 103.8	* 48.1	* 88.3 ± 119.2	* 56.5
	Ø	* 89.4 ± 124.7	* 53.5	* 111.7 ± 138.1	* 61.5

* Significant difference (*p* < .05) between Baseline and Wave 1. SD: standard deviation, m: male, f: female, Ø: all.

**Table 3:** Unorganized sport activity of German children and adolescents.

		MoMo Baseline (2003–2006) *n* = 4528			MoMo Wave 1 (2009–2012) *n* = 3994		
		Unorganized Sport Activity		Playing Outside	Unorganized Sport Activity		Playing Outside
Age (Years)	Sex	Min/Week Mean ± SD	Percentage Engaging	Days/Week Mean ± SD	Min/Week Mean ± SD	Percentage Engaging	Days/Week Mean ± SD
4–5	m	* 54.3 ± 102.0	42.0	6.0 ± 1.5	* 35.9 ± 86.0	37.5	5.9 ± 1.8
	f	* 42.8 ± 103.0	38.9	5.6 ± 1.8	* 35.7 ± 72.0	39.3	5.8 ± 1.6
	Ø	* 48.7 ± 102.6	40.5	5.8 ± 1.6	* 35.7 ± 79.5	38.3	5.8 ± 1.7
6–10	m	* 76.6 ± 123.7	* 53.9	5.4 ± 1.9	* 54.7 ± 112.0	* 41.9	5.5 ± 1.7
	f	* 70.0 ± 111.1	54.4	* 5.1 ± 2.0	* 56.0 ± 93.9	48.1	* 5.6 ± 1.7
	Ø	* 73.3 ± 117.7	* 54.1	* 5.2 ± 2.0	* 55.3 ± 103.4	* 44.9	* 5.6 ± 1.7
11–13	m	* 114.1 ± 169.6	* 60.9	* 4.4 ± 2.2	* 83.0 ± 166.5	* 43.5	* 3.9 ± 2.2
	f	* 83.2 ± 136.4	* 61.4	* 4.3 ± 2.1	* 54.4 ± 111.2	* 41.5	* 3.6 ± 2.2
	Ø	* 99.0 ± 155.0	* 61.1	* 4.4 ± 2.2	* 69.1 ± 142.9	* 42.5	* 3.7 ± 2.2
14–17	m	* 115.5 ± 179.3	* 64.5	* 3.3 ± 2.4	* 86.5 ± 144.1	* 53.8	* 2.3 ± 1.9
	f	* 76.7 ± 129.2	* 55.6	* 2.7 ± 2.4	* 65.6 ± 128.0	* 47.4	* 1.8 ± 1.8
	Ø	* 96.6 ± 158.0	* 60.1	* 3.0 ± 2.4	* 76.2 ± 136.8	* 50.6	* 2.1 ± 1.9
4–17	m	* 94.3 ± 153.3	* 57.3	* 4.6 ± 2.3	* 68.1 ± 134.5	* 45.2	* 4.2 ± 2.4
	f	* 71.4 ± 122.4	* 54.2	4.2 ± 2.4	* 55.9 ± 107.1	* 45.3	4.1 ± 2.5
	Ø	* 83.1 ± 139.6	* 55.8	* 4.4 ± 2.4*	* 62.1 ± 122.0	* 45.2	* 4.1 ± 2.4

* Significant difference (*p* < 0.05) between Baseline and Wave 1. SD: standard deviation, m: male, f: female, Ø: all.
